# Identification and Characterization of Corynaridin, a Novel Linaridin from Corynebacterium lactis

**DOI:** 10.1128/spectrum.01756-22

**Published:** 2022-12-21

**Authors:** Efthimia Pashou, Sebastian J. Reich, Alexander Reiter, Dominik Weixler, Bernhard J. Eikmanns, Marco Oldiges, Christian U. Riedel, Oliver Goldbeck

**Affiliations:** a Institute of Microbiology and Biotechnology, University of Ulm, Ulm, Germany; b Institute of Bio- and Geosciences, IBG-1: Biotechnology, Forschungszentrum Jülich GmbH, Jülich, Germany; c Institute of Biotechnology, RWTH Aachen University, Aachen, Germany; Lerner Research Institute

**Keywords:** *Corynebacterium*, linaridin, antimicrobial peptides, bacteriocins

## Abstract

Genome analysis of Corynebacterium lactis revealed a bacteriocin gene cluster encoding a putative bacteriocin of the linaridin family of ribosomally synthesized and posttranslationally modified peptides (RiPPs). The locus harbors typical linaridin modification enzymes but lacks genes for a decarboxylase and methyltransferase, which is unusual for type B linaridins. Supernatants of Corynebacterium lactis RW3-42 showed antimicrobial activity against Corynebacterium glutamicum. Deletion of the precursor gene *crdA* clearly linked the antimicrobial activity of the producer strain to the identified gene cluster. Following purification, we observed potent activity of the peptide against *Actinobacteria*, mainly other members of the genus *Corynebacterium*, including the pathogenic species Corynebacterium striatum and Corynebacterium amycolatum. Also, low activity against some *Firmicutes* was observed, but there was no activity against Gram-negative species. The peptide is resilient towards heat but sensitive to proteolytic degradation by trypsin and proteinase K. Analysis by mass spectrometry indicates that corynaridin is processed by cleaving off the leader sequence at a conserved motif and posttranslationally modified by dehydration of all threonine and serin residues, resulting in a monoisotopic mass of 3,961.19 Da. Notably, time-kill kinetics and experiments using live biosensors to monitor membrane integrity suggest bactericidal activity that does not involve formation of pores in the cytoplasmic membrane. As *Corynebacterium* species are ubiquitous in nature and include important commensals and pathogens of mammalian organisms, secretion of bacteriocins by species of this genus could be a hitherto neglected trait with high relevance for intra- and interspecies competition and infection.

**IMPORTANCE** Bacteriocins are antimicrobial peptides produced by bacteria to fend off competitors in ecological niches and are considered to be important factors influencing the composition of microbial communities. However, bacteriocin production by bacteria of the genus *Corynebacterium* has been a hitherto neglected trait, although its species are ubiquitous in nature and make up large parts of the microbiome of humans and animals. In this study, we describe and characterize a novel linaridin family bacteriocin from Corynebacterium lactis and show its narrow-spectrum activity, mainly against other actinobacteria. Moreover, we were able to extend the limited knowledge on linaridin bioactivity in general and for the first time describe the bactericidal activity of such a bacteriocin. Interestingly, the peptide, which was named corynaridin, appears bactericidal, but without formation of pores in the bacterial membrane.

## INTRODUCTION

Genome sequencing and bioinformatics have promoted the discovery of ribosomally synthesized, bioactive molecules over the past decades. The heterogeneous group of antimicrobial peptides produced by bacteria, so-called bacteriocins, gained special interest because of their potential use as food preservatives and alternatives to antibiotics (1–5). However, the primary biological function of bacteriocins is to provide the producer with a selective advantage over target organisms in a complex and competitive ecological niche (6, 7).

While bacteriocins have been extensively studied in lactic acid bacteria (LAB), comparably little knowledge is available about production of such compounds by other bacteria (e.g., *Actinobacteria*). Nevertheless, several studies suggest the widespread occurrence of bacteriocin gene clusters (BGCs) in non-LAB species, including the genus *Corynebacterium* (8–11). Species of this genus are widespread in nature, make up one of the largest groups of bacteria in the human and animal skin microbiome, and are also present in food products, including raw milk or cheese (12–14).

Besides toxicogenic *Corynebacterium* species (e.g., Corynebacterium diphtheriae and Corynebacterium ulcerans), many (nondiphtheritic) species of the genus have been described as commensals (13). Being a dominant bacterial group of the human skin microbiome, also nondiphtheritic corynebacteria are regularly found in infectious tissue. However, in most cases they are regarded rather as contamination from surrounding skin than the etiological agent of the infection itself (15). Nevertheless, improved methods to discriminate infection and colonization and an increasing number of reports suggest that some *Corynebacterium* species are important opportunistic pathogens for humans and animals (16). The clinical relevance of nondiphtheritic corynebacteria becomes even more apparent with increasing reports of multidrug-resistant strains, mostly identified in nosocomial environments (17, 18). Species like Corynebacterium striatum and Corynebacterium amycolatum have been described to cause infections in elderly, immunocompromised patients and are associated with chronic wounds (19). Also, recent studies suggest that microbe-microbe interactions of *Corynebacterium* species with other commensals or pathogens like Staphylococcus aureus might influence the behavior and fitness of both species (20). Interestingly, only a very few reports on bacteriocin production in corynebacteria exist (21, 22).

In general, bacteriocins of Gram-positive bacteria can be classified into small (<10 kDa) modified (class I) and unmodified (class II) as well as larger (>10 kDa) heat-labile (class III) peptides/proteins (1). Class I bacteriocins usually contain posttranslational amino acid modifications, such as dehydration, heterocycle formation, glycosylation, methylation, etc., that are often important for their biological activity (23). Thus, class I bacteriocins are also referred to as ribosomally synthesized and posttranslationally modified peptides (RiPPs) (1). Linaridins are a group of RiPPs with an overall linear structure, containing dehydrated amino acids such as dehydrobutyrine (24). So far, only five members of this family have been described in detail: i.e., cypemycin, grisemycin, legonaridin, mononaridin, and salinipeptins (24–30). Nevertheless, *in silico* analyses suggest that linaridin BGCs are widespread in nature and especially in *Actinobacteria* (8). In contrast to lanthipeptides, which also contain dehydrated amino acids (e.g., nisin), linaridin biosynthesis is considered to be essentially different from that of other RiPPs. Modification of the prototypic type A linaridin cypemycin includes dehydration of threonine residues, N-terminal methylation, and C-terminal oxidative decarboxylation of cysteine and subsequent formation of a heterocyclic *S*-[(*Z*)-2-aminovinyl]-d-cysteine (AviCys) moiety (24, 25). N-terminal methylation was shown to be crucial for the activity of cypemycin (28). In contrast, type B linaridin gene clusters do not encode decarboxylases and thus lack C-terminal modification. Instead, genes for so far uncharacterized short-chain oxidoreductases have been identified: e.g., in the gene cluster for the biosynthesis of legonaridin (27).

While extensive studies have been carried out on the structure and chemistry behind their modifications, comparably little is known about the biological functions of linaridins. The antimicrobial activity of cypemycin appears to be limited to Micrococcus luteus (29). Additionally, cypemycin possesses cytotoxic activity against mouse P388 leukemia cells (26, 29). The structurally similar salinipeptins, however, inhibit growth of a Streptococcus pyogenes strain but not that of M. luteus (30). So far, for none of the hitherto described linaridins receptors or mode of action have been proposed.

In this study, we describe the identification and partial characterization of a novel linaridin discovered in Corynebacterium lactis RW3-42, a strain isolated from raw cow’s milk (31).

## RESULTS

### *In silico* analysis of a bacteriocin gene cluster in *C. lactis*.

**FIG 1:**
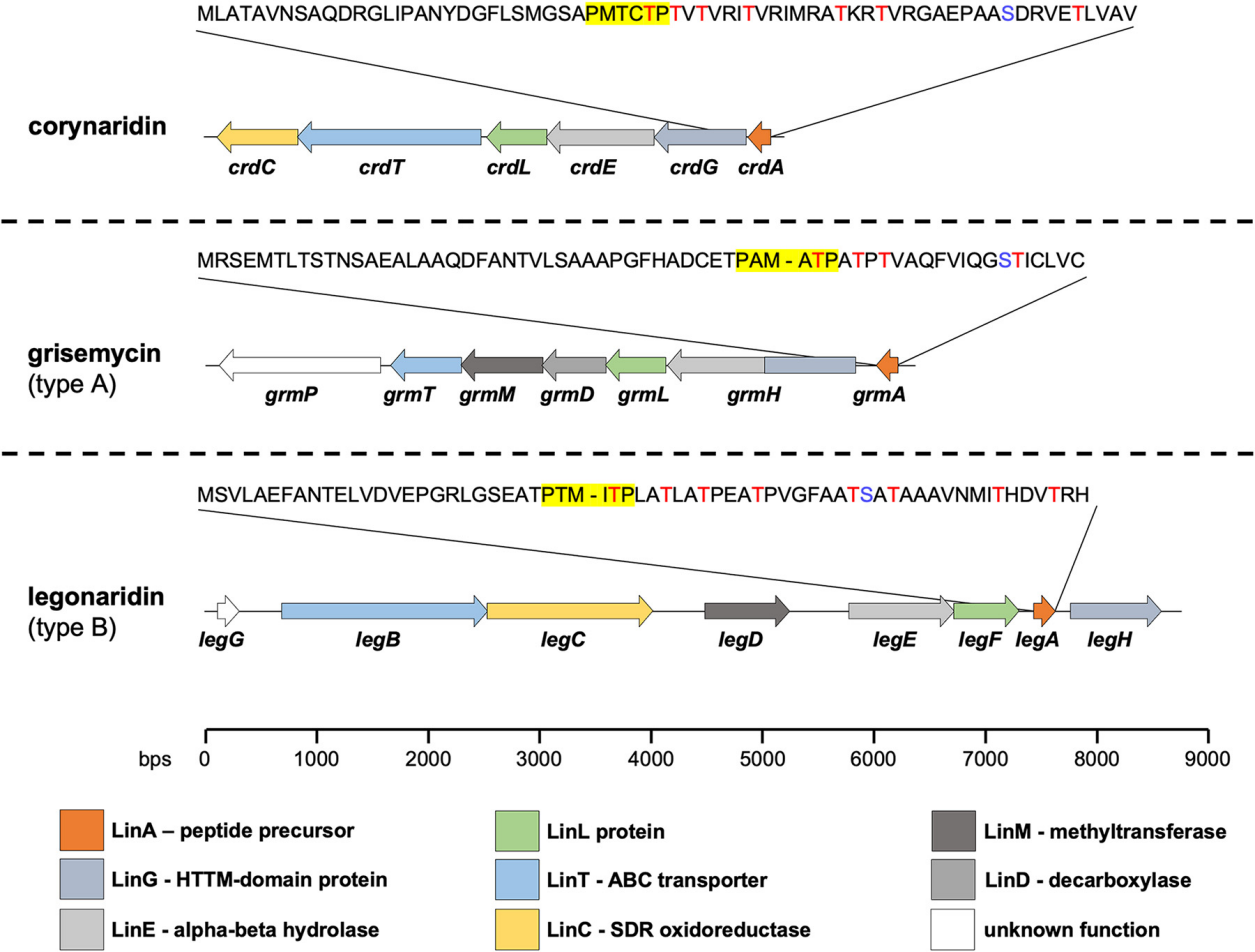
Comparison of the BGC of *C. lactis* RW3-42 with BGCs for other bacteriocins of the linaridin family. Shown is the genetic organization of the corynaridin, grisemycin, and legonaridin BGCs as predicted by BAGEL4 and blastp analyses. The amino acid sequences of the precursor peptides are displayed above the gene cluster. Threonine residues are displayed in red letters. The hexapeptide cleavage site PxxxTP in the precursor peptides is highlighted in yellow. Predicted functions of modification enzymes and their corresponding genes are indicated by a color scheme according to the color key at the bottom of the figure.

*In silico* analyses using the web-based tool BAGEL4 revealed several, yet undescribed bacteriocin gene clusters (BGCs) in the genus *Corynebacterium* (9). In this study, we closely examined one of the predicted BGCs in the genome of *C. lactis* RW2-5 isolated from raw cow’s milk and found an identical cluster in the strain *C. lactis* RW3-42 upon sequencing of the locus (31). The identified BGC consists of six genes, including a gene for a conserved LinL protein homolog (here named *crdL*) that was so far only associated with linaridin biosynthesis and is typically used to identify corresponding gene clusters (Fig. 1) (28). Furthermore, a hypothetical peptide precursor gene (here named *crdA*) was identified that encodes a peptide of 69 amino acids with a conserved hexapeptide cleavage motif, PxxxTP, at positions 29 to 34. The peptide also harbors a high number of threonine residues (seven in total) in its C-terminal part, which are often posttranslationally modified in RiPPs as shown for cypemycin or nisin (Fig. 1) (23).

The deduced amino acid sequences of the other proteins encoded in the BGC were further analyzed using blastp and checked for homologies to enzymes involved in biosynthesis of described members of the linaridin family. Similar to other linaridin BGCs, the *crd* locus contains no gene for a protease involved in removal of the leader peptide but genes for an N-terminal horizontally transferred transmembrane helix domain protein (HTTM domain protein, *crdG*) and an alpha-beta-fold hydrolase (*crdE*) are present (Fig. 1). These putative modification enzymes are assumed to participate in the maturation of linaridins, although their exact mechanism remains unclear (24). Interestingly, no decarboxylase gene but a putative short-chain oxidoreductase (SDR) gene (*crdC*) typical for biosynthetic operons of type B linaridins is part of the BGC. This observation and the absence of a C-terminal cysteine residue in the peptide precursor suggest that the BGC of *C. lactis* encodes a type B linaridin. Notably, we were also unable to identify a gene for a methyltransferase involved in N-terminal methylation, which was described for, e.g., legonaridin (24, 27). The ABC transporter gene (*crdT*) of the BGC is supposedly involved in transport and/or processing of the peptide or immunity of the host (32). Sequence alignments of the enzymes of the *C. lactis* BGC with those of the legonaridin biosynthesis of *Streptomyces* sp. strain CT34 revealed only low overall homologies (22 to 32%) (see Table S2 and Fig. S1 to S6 in the supplemental material). Overall, our *in silico* analyses suggest that the BGC of *C. lactis* RW3-42 encodes a novel type B linaridin lacking N-terminal methylation, for which we propose the designation “corynaridin.”

Furthermore, we analyzed whether the corynaridin precursor exists in other BGCs of *Corynebacterium* species and found a highly similar gene cluster in the genome of C. striatum 1329_CAUR, isolated from the wound of an intensive care unit patient (Fig. S7) (33). The respective peptide precursor shows 84% identity to corynaridin, with a conserved PxxxTP hexapeptide motif and seven putatively modified threonine residues at its N terminus. Interestingly, a previous, extensive bioinformatic analysis of the NCBI genome database identified 561 linaridin BGCs, mainly in *Actinobacteria*, including the ones in *C. lactis* and C. striatum plus four others in *Corynebacterium* spp. (8). Closer examination of these BGCs shows that also in these genomes typical linaridin biosynthesis genes are present, but the overall genetic architectures and precursor peptide sequences are significantly different (Fig. S8 and S9).

### Growth, antimicrobial activity, and kinetics of secretion of corynaridin.

**FIG 2:**
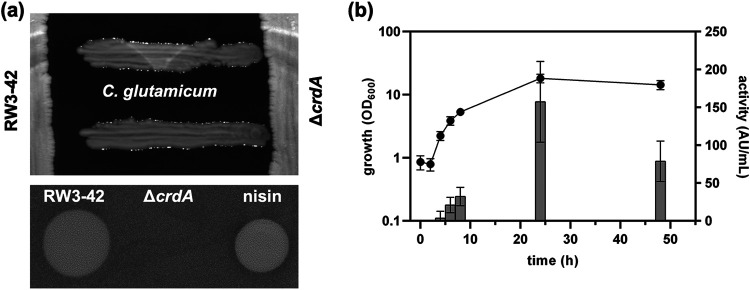
Analysis of the antimicrobial activity produced by *C. lactis* RW3-42. (a) Cross-streak assay (upper panel) and spot-on-lawn assays (lower panel) indicating the secretion of an antimicrobial substance by *C. lactis* RW3-42 compared to the *C. lactis* Δ*crdA* deletion mutant strain using C. glutamicum ATCC 13032 as indicator. Ten microliters of a nisin standard (250 μg/mL) was used as a positive control in the spot-on-lawn assay. (b) Growth (OD_600_) (left *y* axis; black dots) and kinetics of antimicrobial activity (BU per milliliter) (shown as arbitrary units [AU] on the right *y* axis; gray columns) of *C. lactis* RW3-42 in CLI medium containing 1% (wt/vol) glucose. Antimicrobial activity was measured with C. glutamicum ATCC 13032 as an indicator. Values are means and standard deviations from at least six biological replicates.

Since bacteriocins are often most effective against closely related species, we first tested if *C. lactis* RW3-42 is able to inhibit growth of different *Corynebacterium* species. Analysis of its antimicrobial capacity against Corynebacterium glutamicum ATCC 13032 revealed a clear zone of inhibition in cross-streak and spot-on-lawn assays using supernatants of *C. lactis* RW3-42 (Fig. 2a). Also, other *Corynebacterium* species, M. luteus DSM 20030, and Pediococcus acidilactici 347 were inhibited in growth (Table 1 and Table S3). In contrast, no activity of *C. lactis* RW3-42 was detected against Cutibacterium acnes DSM 16379, the other tested *Firmicutes*, or Gram-negative bacteria, like Escherichia coli MG1655 and Pseudomonas fluorescens DSM 50090, in cross-streak assays (Table 1 and Table S3). To verify that the antimicrobial activity is related to the predicted BGC (Fig. 1), we generated a *C. lactis* Δ*crdA* mutant strain with a clean, markerless deletion of *crdA*, encoding the corynaridin precursor. The mutant did not show antimicrobial activity against C. glutamicum ATCC 13032 in cross-streak or spot-on-lawn assays confirming that the antimicrobial activity of *C. lactis* RW3-42 is related to *crdA* (Fig. 2a).

**TABLE 1:** Inhibitory spectrum of *C. lactis* RW3-42 and purified corynaridin assessed with cross-streak and spot-on-lawn assays

Strain	Phylum	Activity^*a*^	
		Cross-streak	RPC fraction^*b*^
Corynebacterium ammoniagenes DSM 20306	*Actinobacteria*	+	+
Corynebacterium amycolatum DSM 6922	*Actinobacteria*	+	+
Corynebacterium canis DSM 45402	*Actinobacteria*	(+)	ND
Corynebacterium casei DSM 44701	*Actinobacteria*	+	+
Corynebacterium efficiens DSM 44549	*Actinobacteria*	+	+
Corynebacterium glutamicum ATCC 13032	*Actinobacteria*	+	+
Corynebacterium lipophiloflavum DSM 44291	*Actinobacteria*	+	+
Corynebacterium striatum DSM 20668	*Actinobacteria*	+	+
Corynebacterium xerosis DSM 20743	*Actinobacteria*	+	+
Cutibacterium acnes DSM 16379	*Actinobacteria*	−	(+)
Micrococcus luteus DSM 20030	*Actinobacteria*	+	+
Bacillus subtilis DSM 402	*Firmicutes*	−	−
Lactobacillus plantarum DSM 1055	*Firmicutes*	−	−
Lactococcus lactis IL1403	*Firmicutes*	−	+
Listeria innocua LMG2785	*Firmicutes*	−	(+)
Listeria monocytogenes EGD-e	*Firmicutes*	−	−
Pediococcus acidilactici 347	*Firmicutes*	+	−
Staphylococcus aureus ATCC 29213	*Firmicutes*	−	−
Staphylococcus epidermidis DSM 3269	*Firmicutes*	−	−
Pseudomonas fluorescens DSM 50090	*Gammaproteobacteria*	−	−
Escherichia coli K-12 MG1655	*Proteobacteria*	−	−

a +, zone of inhibition; (+), diffuse zone of inhibition; −, no zone of inhibition; ND, not determined.

b Ten-microliter RPC fraction.

Growth of *C. lactis* RW3-42 and kinetics of production of the antimicrobial activity in shake flask experiments indicated that the antimicrobial compound is secreted mainly during the exponential growth phase (Fig. 2b). The highest biomass of the strain was observed after 24 h (optical density at 600 nm [OD_600_] = 18 ± 3). Minor antimicrobial activity against C. glutamicum ATCC 13032 was first observed after 4 h and peaked at a maximum of 157 ± 53 bacteriocin units (BU)/mL after 24 h. After 48 h, a reduction of antimicrobial activity occurred, possibly due to degradation or adsorption of the peptide to biomass.

### Purification of corynaridin from supernatants of *C. lactis* RW3-42.

**FIG 3:**
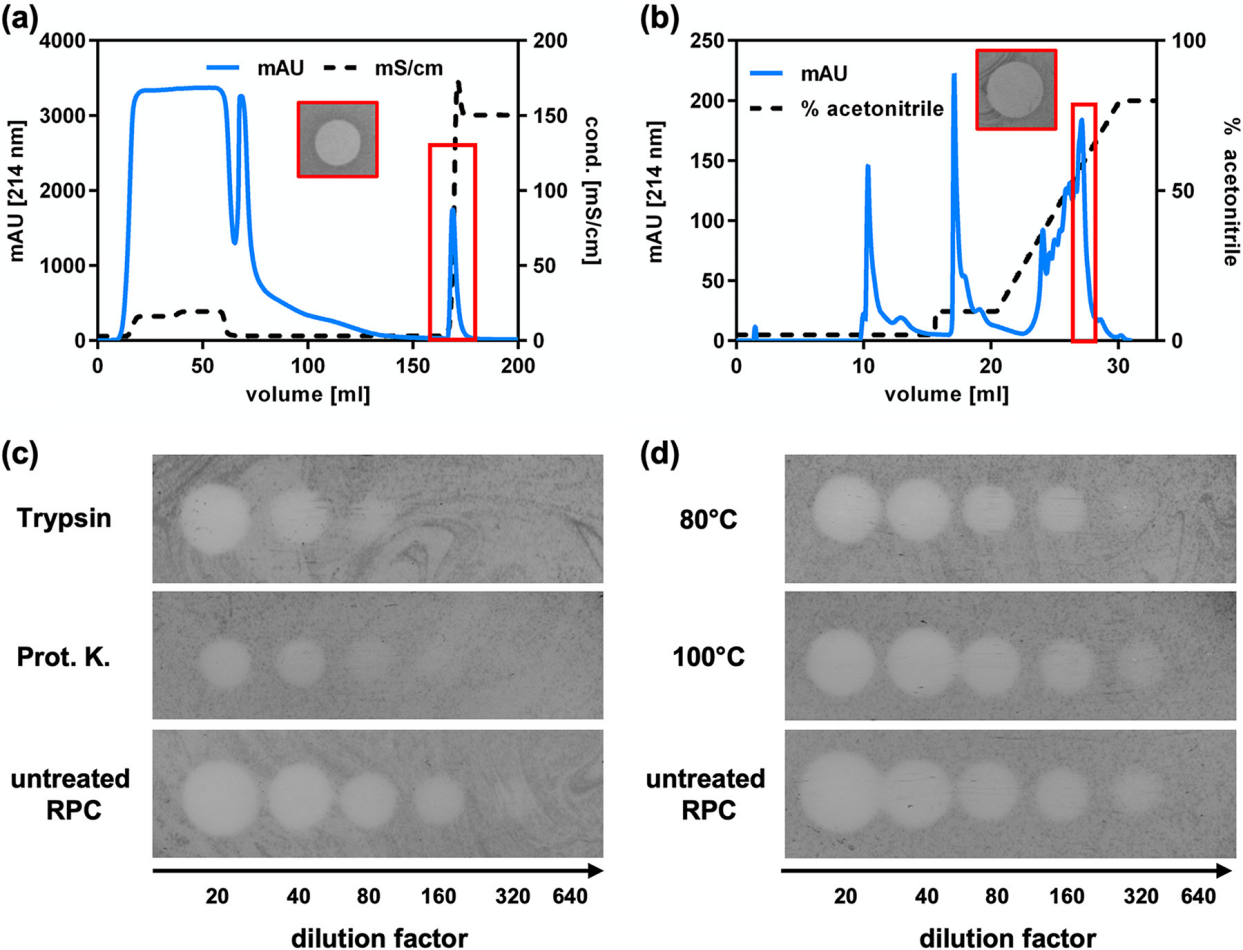
Purification of corynaridin from *C. lactis* RW3-42 supernatants. Supernatant proteins were precipitated by 50% (wt/vol) ammonium sulfate and analyzed by (a) cation-exchange chromatography and (b) reversed-phase chromatography. Absorbance at 214 nm is displayed as blue line, conductivity and acetonitrile concentration are displayed as dashed line. Purified corynaridin (i.e., pooled fractions of the indicated peak of reversed-phase chromatography) was analyzed for stability against (c) proteases and (d) heat treatment. For each spot, a 10-μL sample was used (RPC fraction with 606 μg/mL total protein). One representative experiment is shown for each purification step. Prot. K., proteinase K.

We next sought to purify the secreted antimicrobial compound for further characterization. Supernatants of 1-L cultivations of *C. lactis* RW3-42 were harvested, and proteins were precipitated using ammonium sulfate. The precipitate was resuspended in high-performance liquid chromatography (HPLC)-grade water, with the pH adjusted to 4, and used for cation-exchange chromatography (CIEX) (Fig. 3a). A single peak was observed following onset of elution with high-salt buffer, and the corresponding fractions exhibited activity against C. glutamicum ATCC 13032 in spot-on-lawn assays. Peak fractions were pooled for further purification via reversed-phase chromatography (RPC) using acetonitrile-H_2_O-trifluoroacetic acid (TFA) as the mobile phase (Fig. 3b). A combined step and linear gradient was applied and yielded several peaks, of which only one (at ~56% acetonitrile) showed activity against C. glutamicum ATCC 13032 in spot-on-lawn assays. After removal of acetonitrile and resuspension in HPLC-grade H_2_O, a preparation with high activity (32,000 BU/mL) was obtained.

To assess the physicochemical properties of the purified compound, it was tested for resistance to protease and heat treatment (Fig. 3c and d). While a reduction of activity of the RPC fraction to 8,000 BU/mL (i.e., about 4-fold) was observed after incubation with trypsin or proteinase K, heat treatment at 80°C and 100°C for 10 min had no effect. This indicates that the purified antimicrobial compound is a heat-stable peptide.

### Inhibitory spectrum of purified corynaridin.

**FIG 4:**
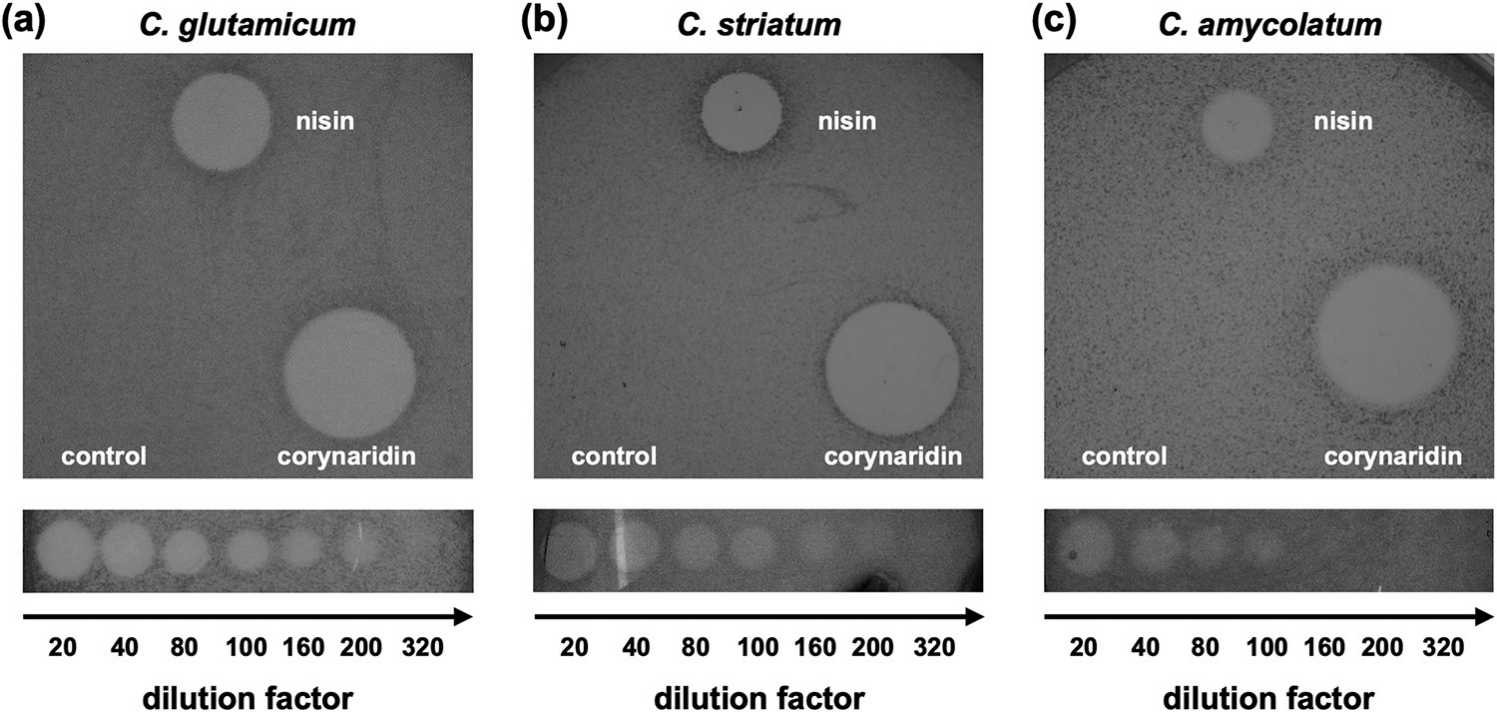
Antimicrobial activity of purified corynaridin against pathogenic corynebacteria. The RPC fraction was used undiluted and in a dilution series against (a) C. glutamicum ATCC 13032, (b) C. striatum DSM 20668, and (c) *C. amycolatum* DSM 6922. For each spot, a 10-μL sample was used (RPC fraction with 606 μg/mL total protein). A nisin standard (250 μg/mL) and HPLC-grade H_2_O (control) were used as positive and negative controls, respectively.

Purified corynaridin was active against all tested *Corynebacterium* species (Table 1 and Table S3) except *C. canis* DSM 45402, which could not be properly analyzed by the spot-on-lawn method. Notably, besides non-pathogenic, environmental and commensal bacteria, including C. glutamicum ATCC 13032 (Fig. 4a), emerging multiresistant pathogens like C. striatum DSM 20668 (Fig. 4b) and *C. amycolatum* DSM 6922 (Fig. 4c) also were inhibited by RPC-purified corynaridin. In case of *P. acidilactici* 347, assays with purified peptide contradicted the cross-streak results as the peptide did not inhibit growth of the strain (Table 1). L. lactis IL-1403 was the only firmicute tested that was effectively inhibited by corynaridin. For *L. innocua* LMG2785 and the actinobacterium *C. acnes* DSM 16379, low levels of inhibition were achieved only at high concentrations (>1,000 μg/mL) of the RPC fraction.

For all other tested Gram-positive bacteria of the phylum *Firmicutes*, including Listeria monocytogenes EGD-e or S. aureus ATCC 29213, or Gram-negative bacteria like E. coli MG1655 or *P. fluorescence* DSM 50090, cross-streak and spot-on-lawn assays equally indicated that these organisms were not inhibited by corynaridin. Collectively, these findings suggest that corynaridin has a rather narrow spectrum of target organisms (Table 1).

### Identification of corynaridin by LC-MS.

**FIG 5:**
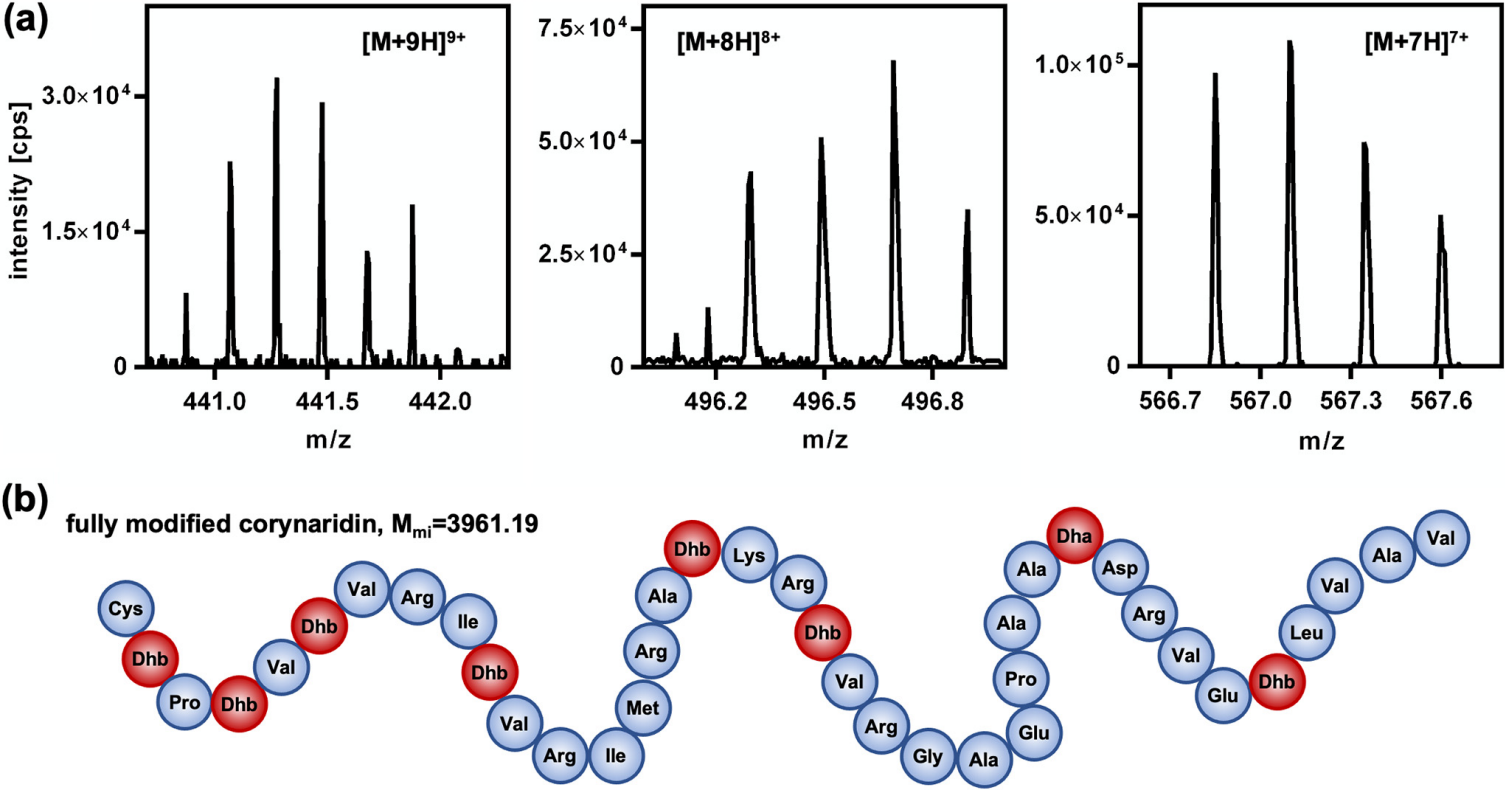
Identification of corynaridin by LC-MS. (a) Representative peaks identified in TOF spectra corresponding to fully modified, processed corynaridin with a monoisotopic mass of 3,961.19 Da carrying seven (right), eight (middle), or nine (left) positive charges (protons); (b) schematic representation of mature corynaridin with dehydration of all threonine/serine residues to dehydrobutyrine (DhB) and dehydroalanine (Dha).

To identify the antimicrobial compound and investigate potential posttranslational modifications, corynaridin was purified from supernatants of *C. lactis* RW3-42 following growth in CLIV minimal medium (see Materials and Methods) with glucose as sole carbon source (Fig. S10a). Under these conditions, slow growth and strong aggregation were observed, making OD_600_ measurements impossible. Nevertheless, activities in supernatants were already observed during the first hours of growth, remained stable after 24 h, and levels were overall comparable to cultivation in complex medium. Supernatants harvested after 24 h of cultivation in CLIV medium were directly applied to the cation-exchange column (Fig. S10b), resulting in a higher initial purity than the previously used protocol. After subsequent RPC purification with a slightly changed protocol, a fraction was obtained that was suitable for mass spectrometry (Fig. S10c and d). The activity of the sample was 40,000 BU/mL, corresponding to 0.5 μg/mL protein (Fig. S10e). The active RPC fraction was analyzed by liquid chromatography-mass spectrometry (LC-MS), yielding rather complex time of flight (TOF) spectra. The linaridins investigated so far contain unusual postranslational modifications and were shown to carry dehydrated threonine residues (dehydrobutyrine). The corynaridin gene cluster contains the genes *crdG*, *crdE*, and *crdL*, which are homologues of the genes suspected to encode the enzymes responsible for threonine dehydration in linaridins (24). The primary amino acid sequence of processed corynaridin (i.e., following cleavage of the leader sequence) also contains a serine residue at position 29 that may be dehydrated in lantibiotics to dehydroalanine as shown for nisin (22). Not taking into account positional variations of dehydration and assuming processing by cleavage of the leader sequence, corynaridin may carry up to 8 dehydrated threonines/serines, resulting in 9 distinct monoisotopic masses, and each of these variants may be present in various isotopic states and carrying additional charges (protons). To identify corynaridin, the TOF spectra were filtered for signals with a minimum peak intensity of 10^3^ cps for at least three charged peptide species with at least four isotopes. The vast majority of signals in the time-resolved spectra that matched these criteria corresponded to fully modified, processed corynaridin (dehydration of all 8 threonine/serine residues) with a monoisotopic mass of 3,961.19 Da (Fig. 5 and Table S4). However, it has to be noted that some of the signals that passed the thresholds of filtering correspond to peptide variants with lower numbers of dehydrated threonine/serine residues (Table S4).

### Corynaridin exhibits bactericidal activity.

**FIG 6:**
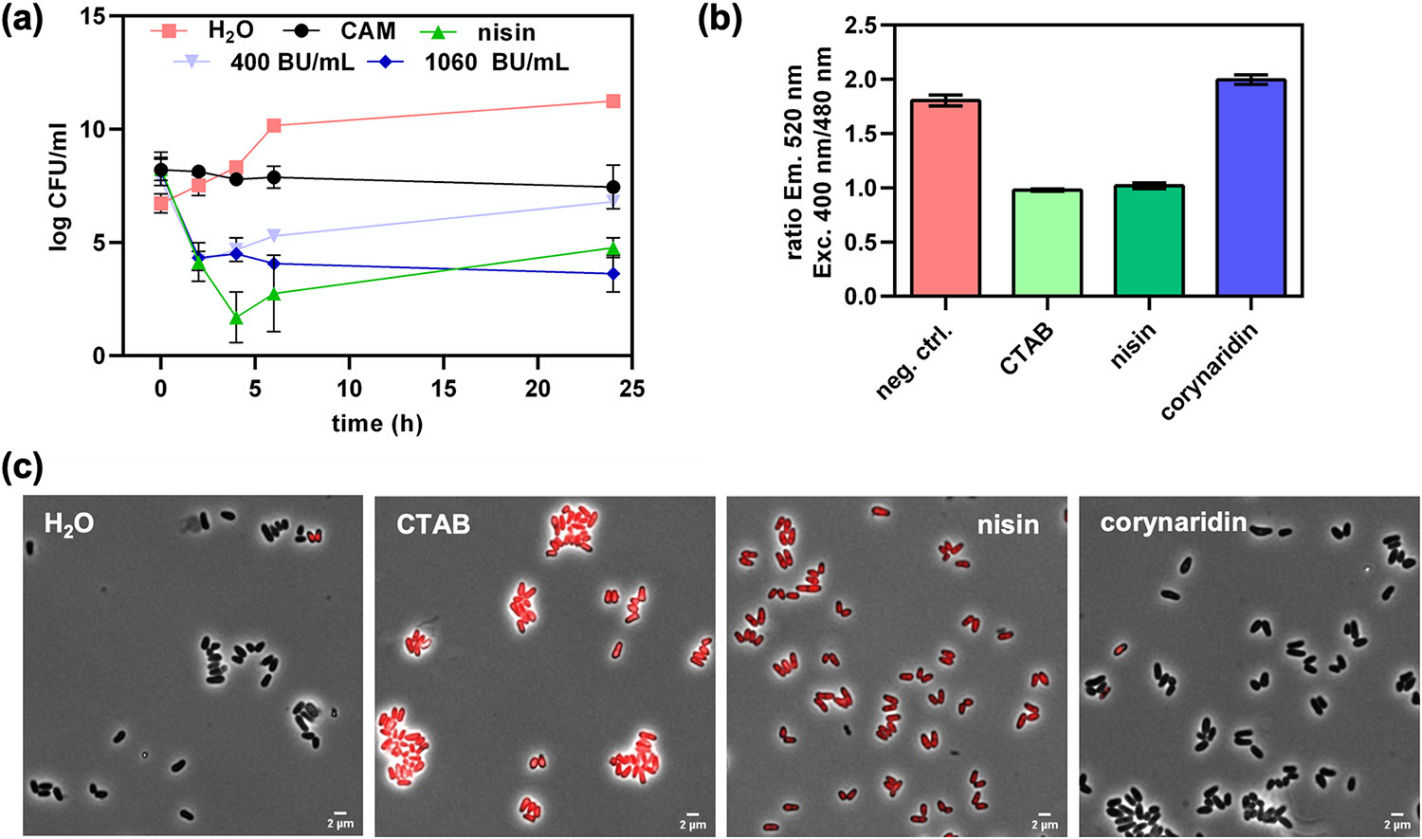
Bactericidal activity of corynaridin. (a) Time-kill assays with C. glutamicum ATCC 13032 as indicator, 1.25 μg/mL nisin, 6.5 μg/mL chloramphenicol, and H_2_O as controls, and corynaridin (RPC fractions at 400 and 1060 BU/mL). (b) Fluorescence intensity (emission at 50 nm) ratios at 400 and 480 nm of excitation for H_2_O (negative control [neg. ctrl.]), with CTAB, nisin, and corynaridin added to C. glutamicum ATCC 13032/pPB-pHin2*^Cg^* cells to monitor membrane damage. Values are means and standard deviations from at least three biological replicates. (c) Fluorescence microscopic pictures merged with phase-contrast images (×63) of propidium iodide-stained C. glutamicum ATCC 13032 cells after treatment with H_2_O, 0.05% CTAB, 1.25 μg/mL nisin, and 4,000-BU/mL RPC fraction containing corynaridin.

To elucidate whether corynaridin shows bactericidal or bacteriostatic activity, we employed time-kill assays with C. glutamicum ATCC 13032 as an indicator. As controls for bacteriostatic and bactericidal compounds, we used the antibiotic chloramphenicol and the pore-forming class I bacteriocin nisin, respectively (Fig. 6a). Addition of the vehicle H_2_O to C. glutamicum cultures had no effect on cell viability and CFU/mL steadily increased over the course of the experiment. In contrast, cultures treated with 1.25 μg/mL nisin showed a >4-log-reduced number of CFU/mL after 2 h of cultivation as a consequence of the bactericidal effect of the peptide (Fig. 6a). Addition of 6.5 μg/mL (bacteriostatic) chloramphenicol resulted in stable levels of CFU per milliliter throughout the experiment. Corynaridin-containing RPC fractions at 400 BU/mL and 1,060 BU/mL decreased the CFU per milliliter of the indicator after 2 h by 4 orders of magnitude, indicating bactericidal activity of the peptide. While cultures treated with the high concentration remained at low levels of CFU/mL, those treated with the lower concentrations showed slightly increased CFU/mL after 24 h, similar to nisin-treated samples, indicating a bactericidal mode of action of corynaridin.

As bactericidal activity of bacteriocins is often related to the formation of pores in the bacterial membrane, we tested whether corynaridin causes membrane damage using a fluorescence-based whole-cell biosensor assay with C. glutamicum ATCC 13032/pPB-pHin^Cg^ (34). The strain harbors a plasmid for expression of pHluorin2, a fluorescent protein with a pH-dependent, bimodal excitation spectrum which is highly suitable to determine bacteriocin-driven pore formation (35). As expected, the detergent CTAB and pore-forming nisin led to reduced fluorescence ratios for C. glutamicum ATCC 13032/pPB-pHin^Cg^, suggesting membrane damage (Fig. 6b). In contrast, addition of the RPC-purified corynaridin preparation at concentrations up to 10,000 BU/mL did not result in a change of fluorescence ratios. These results were further supported by fluorescence microscopy of propidium iodide-stained bacteria, which provided no evidence for compromised integrity of the bacteria (Fig. 6c). Thus, our data strongly suggest that unlike nisin and other bactericidal bacteriocins, corynaridin does not act by damaging the membrane of target cells under the tested conditions but by an as yet unknown bactericidal mode of action.

## DISCUSSION

In this study, we identified a putative linaridin BGC in the genome of *C. lactis* RW3-42, which was isolated from raw cow’s milk (31). Deletion of the peptide precursor gene *crdA* in the genome of *C. lactis* RW3-42 completely abolished antimicrobial activity of the strain and thus confirmed the identified BGC as locus for the biosynthesis of an antimicrobial compound, which we designated corynaridin. Further *in silico* analyses revealed several adjacent genes for putative modification enzymes that are typically associated with the linaridin family of RiPPs (24). The absence of a decarboxylase gene in the *C. lactis* BGC suggests that the peptide is a type B linaridin, such as legonaridin or mononaridin (27, 36). In contrast, the corynaridin gene cluster also lacks a methyltransferase gene, which makes it unique among the hitherto described BGCs coding for peptides of the linaridin family. Mass spectrometry of the purified peptide revealed that corynaridin is processed at the predicted hexapeptide motif and displays a mass of 3,961.19 Da with all seven threonines as well as the serine dehydrated to dehydrobutyrines and dehydroalanine, respectively. The LC-MS data also suggest that the peptide is not dimethylated at its N terminus like other linardins (24). This is in line with the lack of a *linM* homologue for a methyltransferase in the corynaridin gene cluster. Interestingly, we also observed variants of the mature peptide with fewer threonine/serine residues dehydrated. Whether these peptides are artifacts of the sample preparation or represent true biological variation of the same precursor peptide that may also have different activity and spectra of target organisms needs to be addressed in future studies.

Purified corynaridin was stable at temperatures up to 100°C but lost activity after incubation with proteinase K or trypsin. Heat stability is a favorable trait of many bacteriocins and was also shown for, e.g., nisin (37). Interestingly, corynaridin obviously has a bactericidal mode of action against C. glutamicum ATCC 13032 in liquid cultivations but did not lead to the formation of pores as described for other class I bacteriocins such as nisin (38). As to our knowledge no receptor has been identified for the linaridins described so far, it remains to be investigated how these peptides exert their selective antimicrobial activity and, in some cases, cytotoxic activity against cancer cell lines (26).

Characterization of corynaridin revealed antimicrobial activity against several other actinobacteria comprising pathogenic and commensal *Corynebacterium* species as well as M. luteus and *C. acnes*, but it showed only low or no activity against a selection of *Firmicutes* and Gram-negative bacteria. *C. lactis* and other *Corynebacterium* species are frequently found in raw milk (14, 31), and some isolates of Corynebacterium bovis and *C. amycolatum* are causative agents of mastitis in dairy cows (39, 40). *C. lactis* was eventually linked to infections in companion animals, but it is not considered a pathogen so far and the determinants of infection remain unclear (41, 42). Interestingly, a putative BGC similar to that of corynaridin exists in the genome of an isolate of C. striatum from an intensive care unit (33). C. striatum is a ubiquitous species and part of the human microbiome but can also lead to infections in immunocompromised patients (43, 44). Moreover, Georgiou et al. predicted linaridin BGCs in six *Corynebacterium* spp., including the ones in *C. lactis* and C. striatum but also in a C. diphtheriae strain (8). These bacteria are not only phylogenetically related but might also be competitors in their respective habitats. Thus, secretion of bacteriocins by corynebacteria might be a hitherto largely neglected mechanism for intraspecies competition as described for other bacterial groups (e.g., lactic acid bacteria) and might have an impact on the composition of (actino)bacterial communities in general (20, 45).

In conclusion, we identified a novel, heat-stable bacteriocin produced by *C. lactis* RW3-42 that exerts narrow-spectrum bactericidal activity against other *Actinobacteria*—mainly *Corynebacterium* species. Our experiments suggest that corynaridin has a yet undescribed, bactericidal mode of action that does not involve pore formation. As other linaridins also showed growth-suppressing activity against cancer cell lines, they might be promising candidates for biotechnological exploitation.

## MATERIALS AND METHODS

### Bacterial strains and cultivation conditions.

The bacterial strains used in this study (Table 2) were cultivated in GM17 medium (L. lactis IL-1403), MRS medium (Lactiplantibacillus plantarum DSM1055, *P. acidilactici* 347) or brain heart infusion (BHI) medium (all others) at 37°C (E. coli MG1655, Listeria innocua LMG2785, L. monocytogenes EGD-e, S. aureus ATCC 29213, Staphylococcus epidermidis DSM 3269, *C. acnes* DSM 16379, *C. canis* DSM 45402, Corynebacterium efficiens DSM 44569, *C. amycolatum* DSM 6922, C. striatum DSM 20668, Corynebacterium lipophiloflavum DSM 44291) for the indicated bacteria listed in parentheses or 30°C for all others, respectively. Solidified medium was prepared by addition of 16 g agar per L to the medium. For growth characterization and production, *C. lactis* strains were cultivated in Corynebacterium lactis (CL) medium I (CLI) (21 g/L MOPS [morpholinepropanesulfonic acid], 1 g/L K_2_HPO_4_, 1 g/L KH_2_PO_4_, 16 g/L tryptone, 10 g/L yeast extract, 0.25 g/L MgSO_4_, 0.01 g/L CaCl_2_, 0.2 mg/L biotin [pH adjusted to 7]) or CLIV [21 g/L MOPS, 1 g/L KH_2_PO_4_, 1 g/L K_2_HPO_4_, 10 g/L (NH_4_)_2_SO_4_, 0.1 g/L glucose, 0.2% Tween 80, SL10 trace elements, 0.2 mg/L biotin 0.01 g/L CaCl_2_, 0.25 g/L MgSO_4_ (pH adjusted to 7)]. For time-kill kinetics, 2× TY medium (16 g/L tryptone, 10 g/L yeast extract, 5 g/L NaCl) was used for the cultivation of C. glutamicum ATCC 13032. Cultivation medium of strains carrying plasmids additionally contained kanamycin (25 μg/mL) or chloramphenicol (15 μg/mL). Growth was monitored photometrically by measuring the optical density at 600 nm (OD_600_) at the indicated time points.

**TABLE 2:** Bacterial strains and plasmids used in this study

Species or plasmid	Strain or plasmid characteristic	Source or reference^*a*^
Species		
Bacillus subtilis	DSM 402	DSMZ
Corynebacterium lactis	RW3-42	31
	RW3-42 Δ*crdA*	This study
Corynebacterium ammoniagenes	DSM 20306	DSMZ
Corynebacterium amycolatum	DSM 6922	DSMZ
Corynebacterium canis	DSM 45402	DSMZ
Corynebacterium casei	DSM 44701	DSMZ
Corynebacterium efficiens	DSM 44549	DSMZ
Corynebacterium glutamicum	ATCC 13032	ATCC
Corynebacterium lipophiloflavum	DSM 44291	DSMZ
Corynebacterium striatum	DSM 20668	DSMZ
Corynebacterium xerosis	DSM 20743	DSMZ
Cutibacterium acnes	DSM 16379	DSMZ
Escherichia coli	MG1655	52
Lactiplantibacillus plantarum	DSM 1055	DSMZ
Lactococcus lactis	IL1403	53
Listeria innocua	LMG2785	54
Listeria monocytogenes	EGD-e	55
Micrococcus luteus	DSM 20030	56
Pediococcus acidilactici	347	57
Pseudomonas fluorescens	DSM 50090	DSMZ
Staphylococcus aureus	ATCC 29213	ATCC
Staphylococcus epidermidis	DSM 3269	58
Plasmids		
pk19mobsacB	Km^r^ mobilizable (*oriT*); *oriV*	59
pk19mobsacB_del-crdA	pK19 derivative with 750-bp flanking regions of *crdA* gene of *C. lactis* RW3-42 genome	This study
pPB-pHin2*^Cg^*	pPBEx2 derivative with codon-optimized pHluorin2 gene under control of P_tuf_; Km^r^; pMB1 origin; pBL1 origin	34
pBAD33	Cm^r^; pACYC184/p15A origin; *araC*	60
pBAD33_crd	pBAD33 derivative with *crd* gene cluster under control of P_BAD_	This study

a DSMZ, Deutsche Sammlung von Mikroorganismen und Zellkulturen; ATCC, American Type Culture Collection.

### Molecular biology procedures.

Construction of pk19mobsacB_del-crdA and pBAD33_crd was carried out with standard reagents and according to protocols of the manufacturers. Oligonucleotides are listed in Table S1 in the supplemental material and were obtained from Eurofins Genomics (Ebersberg, Germany). PCR was performed in a C100 thermocycler (Bio-Rad Laboratories, Munich, Germany) using Q5 high-fidelity polymerase (New England Biolabs, Ipswich, USA) and nucleotides from Bio-Budget (Krefeld, Germany). The up- and downstream regions of the *crdA* gene and the *crd* gene cluster were amplified using oligonucleotides creating overlapping ends for Gibson assembly. The empty vector pK19mobsacB was linearized by restriction endonucleases EcoRI and SalI. The empty vector pBAD33 was linearized by restriction endonucleases KpnI and SalI. The final plasmids were verified by sequencing (Eurofins Genomics, Ebersberg, Germany). All plasmids and their relevant characteristics are listed in Table 2. For transformation, *C. lactis* was rendered electrocompetent and transformed as described previously for C. glutamicum (46).

### *In silico* analyses.

Prediction of bacteriocin gene clusters (BGCs) in the genome of *C. lactis* RW2-5 was carried out using BAGEL4 (47). Subsequently, blastp analysis (48) was performed using the deduced proteins with standard parameters (BLOSUM62; gap existence costs, 11; gap extension cost, 1) and assigned to putative functions based on sequence similarities with published linaridin biosynthesis genes (24). The BGC of *C. lactis* RW3-42 was PCR amplified and cloned into pBAD33, followed by sequencing (Eurofins Genomics, Ebersberg, Germany). The resulting sequences were then aligned to the BGCs of *C. lactis* RW2-5 and the legonaridin biosynthesis genes. Sequence alignments were carried out using ClustalW (49) and visualized using Jalview (50).

### Purification of corynaridin.

For purification of corynaridin, proteins of supernatants (1 L) collected after 24 h of cultivation of *C. lactis* RW3-42 in CLI containing 1% (wt/vol) glucose were precipitated using ammonium sulfate (50% [wt/vol] saturation) at 4°C overnight (16 h). The precipitate was collected by centrifugation (60 min, 10,000 × *g*, 4°C) and resuspended in 50 mL H_2_O, and pH was adjusted to 4.0 using 2 M HCl. An additional centrifugation step (10 min, 10,000 × *g*, 4°C) was performed to remove insoluble particles. All of the following chromatographic steps were carried out with an ÄKTA pure chromatography system (Cytiva). The solution containing the peptide was applied to a HiPrep SP FF 16/10 column (GE Healthcare Life Sciences) equilibrated with 20 mM sodium phosphate buffer at pH 3.9. Unbound proteins were washed out by 5 column volumes (CVs) of 20 mM sodium phosphate buffer at pH 6.9 (buffer A). The remaining bound peptides/proteins were then eluted in with 5 CVs of 20 mM sodium phosphate buffer at pH 6.9 with 2 M NaCl (buffer B). The eluate fractions containing the bacteriocin were identified by activity assays (described below) and directly applied to reversed-phase chromatography (RPC) using a 1-mL Resource RPC column (Cytiva) or stored at −20°C until further use. To remove weakly bound proteins, a washing step was carried out with 5 CVs of 2% acetonitrile in H_2_O plus 0.065% TFA. Elution was performed with an initial step to 15% acetonitrile for 5 CVs followed by a linear gradient up to 80% acetonitrile. To obtain corynaridin in a higher purity for liquid chromatography-mass spectrometry (LC-MS), *C. lactis* was cultivated in CLIV medium with 1% (wt/vol) glucose for 24 h and supernatants were harvested as described above. After adjustment of the pH of the solution to 4 and filtration through a 0.2-μm-pore filter, the solution was directly applied to a HiPrep SP FF 16/10 column (GE Healthcare Life Sciences) equilibrated with H_2_O at pH 4.3 (adjusted with HCl). Unbound proteins were washed out by 2 CVs of buffer A followed by a step to 2% buffer B for 3 CVs. Elution started with a step to 5% buffer B followed by a step to 30% buffer B and a final elution at 100% buffer B. Fractions with antimicrobial activity were identified by activity assays (see below), dried using a vacuum concentrator at 60°C (Eppendorf, Hamburg, Germany), and resuspended in HPLC-grade H_2_O. Protein concentrations of the purification fractions were estimated using the Pierce bicinchoninic acid (BCA) protein assay kit (Thermo Fisher Scientific) according to the manufacturer‘s protocol.

### Radial streak.

Bioprospecting for antimicrobial activity was performed using a modified cross-streak method (51). Briefly, *C. lactis* RW3-42 was inoculated from an overnight culture as a single streak with an inoculation loop in the center of an BHI medim agar plate and incubated aerobically for at least 3 days at 30°C. Indicator bacteria (Table 2) were cultivated overnight in 5 mL BHI medium and streaked in a line from the border of the plate toward *C. lactis*. The plates were then incubated for 24 to 48 h at 30 to 37°C, depending on the indicator bacteria. In the case of *C. acnes*, incubation was carried out in an anaerobic jar (Merck KGaA, Darmstadt, Germany) containing an AnaeroGen anaerobic incubation system (Thermo Fisher Scientific).

### Determination of antimicrobial activity.

Antimicrobial activity was determined using a spot-on-lawn assay. Overnight cultures of the respective strains were inoculated with an OD_600_ of 0.01 into hand-warmed agar medium (16 g/L agar) and poured into sterile petri dishes. After solidification, surfaces of agar plates were air dried at room temperature under a sterile hood. Supernatants and purification fractions were serially diluted and spotted onto agar plates. Plates were incubated under the preferred conditions of the embedded bacteria until growth was visible. Volumetric bacteriocin activity (bacteriocin units [BU] per milliliter) was determined by dividing the last dilution factor resulting in a visible zone of inhibition by the volume spotted.

### Liquid chromatography-mass spectrometry.

Following RPC, purified protein fractions were concentrated to ~1 mg mL^−1^ with LC-MS-grade H_2_O prior to LC-MS measurements. LC-MS was performed on an Agilent 1260 Infinity system (Agilent Technologies, Waldbronn, Germany) coupled to a quadrupole time-of-flight (QTOF) mass spectrometer (TripleTOF6600; AB Sciex, Darmstadt, Germany). LC was performed with an Ascentis Express peptide ES-C_18_, 2.7-μm HPLC column (53307-U; Merck, Darmstadt, Germany) with a flow rate of 200 μL min^−1^ and the mobile phases A (0.1% formic acid in water) and B (acetonitrile). Ultraperformance liquid chromatography (UPLC)-MS-grade 0.1% formic acid in H_2_O and acetonitrile were obtained from Biosolve BV (Valkenswaard, Netherlands). LC-MS-grade H_2_O was obtained from a Milli-Q water purification system (Merck Millipore, Burlington, MA, USA). The elution gradient was as follows: 0–70 min: linear gradient from 3 to 40% B, 70–78 min: constant 40% B, 78–79 min: step from 40 to 60% B, 79–89 min: constant 60% B, 89–90 min: step from 60 to 3% B followed by a 12-min equilibration time between injections. The column temperature was set to 21°C and injection volume to 10 μL. MS was conducted with a TurboV ion source operated in positive-ionization mode. The ion spray voltage was set to 5.5 kV, source temperature to 450°C, curtain gas to 35 lb/in^2^, and the support gases GS1/GS2 to 50 lb/in^2^/50 lb/in^2^. All gases were nitrogen. The QTOF mass spectrometer was operated in TOF scan mode with a dwell time of 250 ms. The declustering potential was set to 120 V and mass tolerance to 25 ppm.

Acquired mass spectra were analyzed with Python 3.9.7 and the packages pyopenms 2.7.0, pandas 1.3.4, numpy 1.21.3, pathlib 1.0.1 and matplotlib 3.4.3. TOF spectra were centroided with pyopenms and subsequently filtered for potential peptide mass-to-charge (*m/z*) ratios. Corynaridin peptide variants with different numbers of dehydrated serine or threonine residues were considered identified when *m/z* peaks had a minimum signal intensity of 10^3^ cps for at least three charged peptide species with at least four isotopes.

### Time-kill kinetics.

Fresh overnight cultures of C. glutamicum ATCC 13032 were used to inoculate 5 mL 2× TY in glass tubes with a starting OD_600_ of 0.5 (i.e., ~10^7^ CFU/mL). Samples of interest were added at the indicated concentrations prior to inoculation of the medium. Cultures were then incubated on a rotary shaker at 130 rpm for 24 h at 30°C. Samples were collected at the indicated time points, diluted (10^−1^ to 10^−8^), and plated on 2× TY agar. CFU per milliliter were determined after 24 to 48 h of incubation of the plates at 30°C by counting the colonies for the respective dilution.

### pHluorin assay.

For detection of membrane damage, a pHluorin assay was conducted as described earlier (34). In particular, a 5-mL BHI overnight culture containing kanamycin (50 μg/mL) of the sensor strain C. glutamicum ATCC 13032/pPB-pHin2*^Cg^* was harvested by centrifugation and resuspended to an OD_600_ of 3 in Listeria minimal buffer [LMB: 100 mM MOPS, 4.82 mM KH_2_PO_4_, 11.52 mM Na_2_HPO_4_, 1.7 mM MgSO4, 0.6 g/L (NH_4_)_2_SO_4_, 55 mM glucose (pH 6.2)]. Serial 2-fold dilutions of samples were prepared in black 96-well microtiter plates (Sarsted, Nümbrecht, Germany) with a final volume of 100 μL in each well. Subsequently, 100 μL of the sensor strain suspension was added and the plate was incubated at room temperature in the dark for 30 min. Then, pHluorin2 fluorescence was measured at 520 nm with excitation at the distinct maxima at 400 and 480 nm using an infinite M200 plate reader (Tecan, Männedorf, Switzerland).

### Fluorescence microscopy.

A fresh culture of C. glutamicum ATCC 13032 was washed once in phosphate-buffered saline (PBS), and bacteria were resuspended in saline (0.9% [wt/vol] NaCl) at an OD_600_ of 1. An 87.5-μL concentration of the cell suspension was mixed with nisin, cetyltrimethylammonium bromide (CTAB), H_2_O, or the RPC fraction containing corynaridin to the indicated concentrations and incubated for 10 to 30 min in the dark. Then, the bacteria were stained using 12.5 μL propidium iodide (25 μg/mL) (Invitrogen, Darmstadt, Germany) and again incubated for 15 min in the dark. Samples were imaged using a Axio Observer Z1 (Zeiss, Oberkochen, Germany) in bright-field and fluorescence mode with a filter set for propidium iodide (excitation at 575 to 625 nm, emission at 660 to 710 nm). Images were acquired with a 63× lens objective and analyzed using the Zen software (version 2.3 SP1; Zeiss).
