# Supplementary material for: Identification and Characterization of Corynaridin, a Novel Linaridin from Corynebacterium lactis
Source: Microbiol Spectr. 2022 Dec 21;11(1):e01756-22. doi: 10.1128/spectrum.01756-22 (PMC9927463; doi:10.1128/spectrum.01756-22)
Supplement: Supplemental file 1 — Supplemental material. Download spectrum.01756-22-s0001.pdf, PDF file, 2.2 MB [file spectrum.01756-22-s0001.pdf]

-Supplementary data-

Identification and Characterization of Corynaridin, a Novel Linaridin from Corynebacterium lactis

Efthimia Pashou[1], Sebastian J. Reich[1], Alexander Reiter[2,3], Dominik Weixler[1], Bernhard J. Eikmanns[1], Marco Oldiges[2,3], Christian U. Riedel[1#], and Oliver Goldbeck[1#]

[1] Institute of Microbiology and Biotechnology, University of Ulm, Ulm, Germany

[2] Institute of Bio- and Geosciences, IBG-1: Biotechnology, Forschungszentrum Jülich GmbH, 52425 Jülich, Germany

[3] Institute of Biotechnology, RWTH Aachen University, 52062 Aachen, Germany

# Corresponding authors.

E-mail:

oliver.goldbeck@uni-ulm.de, christian.riedel@uni-ulm.de

Address:

Institute of Microbiology and Biotechnology

University of Ulm

Albert-Einstein-Allee 11

89081 Ulm

Germany

Table S1: List of oligonucleotides used in this study.

Primer	Sequence (5' → 3')	Purpose
pk19_fw	GTATGTTGTGTGGAATTGTGAG	Control primer flanking MCS pK19mobsacB
pk19_rev	CAGGGTTTTCCCAGTCACGACG	
Do_crdA_fwd	CAAGCTTGCATGCCTGCAGGAAGAGGAACATTTGCCGC	Amplification of downstream region of *crdA*
Do_crdA_rev	GGTAGTCAGTTAGGTACTAACTAAAAATGATTGACG	
Up_crdA_fwd	TTAGTACCTAACTGACTACCTTCCATTCTTAGG	Amplification of upstream region of *crdA*
Up_crdA_rev	TTGTAAAACGACGGCCAGTGGAGGAAGAATGCCGGAGAAAC	
CorePeptide-HTHHyd-LinL_fwd	AGCGAATTCGAGCTCGGTACCCTAGATTCTAAGATAAGGAGG TAAATAATGCTCGCTACTGCAGTTAATTC	Contruction of pBAD33_crd
CorePeptide-HTHHyd-LinL_rev	TCAGTTCAGGCTCTTGCCGAGGAATGGG	Contruction of pBAD33_crd
ABC-SDR_fwd	TCGGCAAGAGCCTGAACTGAGAAAATTGAC	Contruction of pBAD33_crd
ABC-SDR_rev	CAAGCTTGCATGCCTGCAGGGGGAAATTACTCTGCTAAAAC	Contruction of pBAD33_crd
pBAD33_fw	ATTATTTGCACGGCGTCA	sequencing of *crd*-locus
pBAD33_rev	GTTTTATCAGACCGCTTCT	sequencing of *crd*-locus
S1	GAGCTCGGTACCCTAGATTC	sequencing of *crd*-locus
S2	GTGGCTGTCTAGGTACTAAC	sequencing of *crd*-locus
S3	TCCACCAATCGGCTTAAAGG	sequencing of *crd*-locus
S4	GAGGTCGTGTTCGGGTCTTG	sequencing of *crd*-locus
S5	CGCAATGACAGCCTTTAACC	sequencing of *crd*-locus
S6	GATTCAACCGAGAGCAGTTC	sequencing of *crd*-locus
S7	GTATCGGTCGGGCAGTTGTG	sequencing of *crd*-locus

Table S2: List of open reading frames contained in the *C. lactis* RW3-42 corynaridin gene cluster, the respective number of amino acids, putative TM-helices and homology to legonaridin biosynthesis proteins.

orf[1]	amino acids	TM-helices	legonaridin homolog	amino acid identity (%) to legonaridin homologs
crdA	69	-	*legA*	25
crdG	275	1	*legH*	32
crdE	324	-	*legE*	21
crdL	182	-	*legF*	26
crdT	550	6	*legB*	31
crdC	243	-	*legC*	25

[1]orf = open reading frame

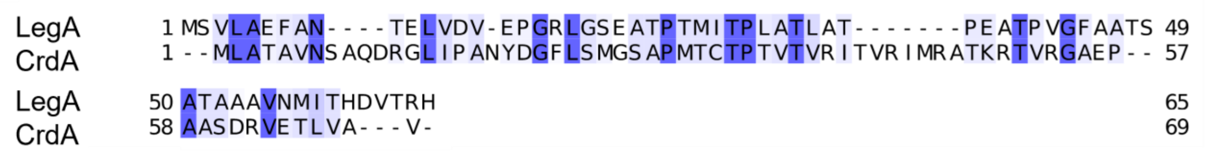

Figure S1: CLUSTALW sequence alignment of LegA (Legonaridin) from *Streptomyces* sp. CT34 and CrdA (corynaridin) from *C. lactis* RW3-42.

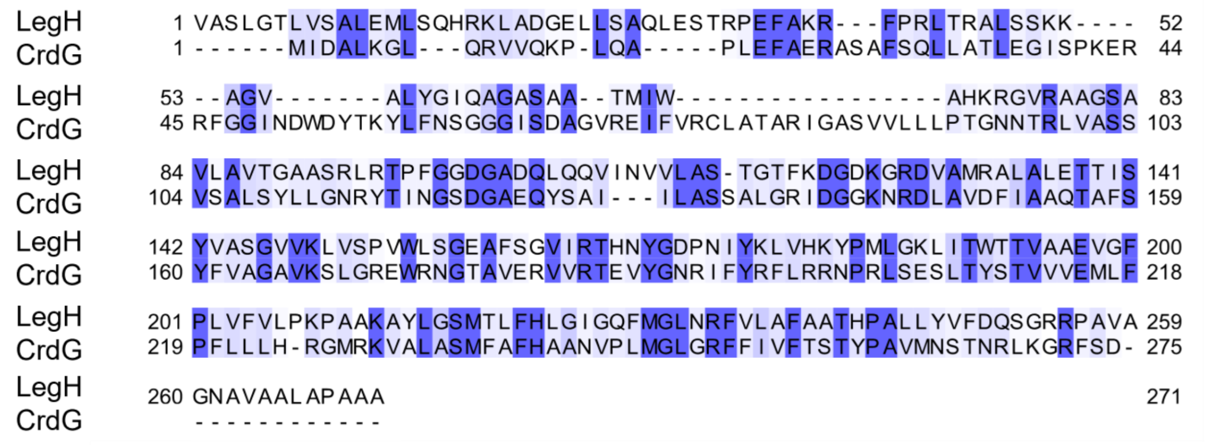

Figure S2: CLUSTALW sequence alignment of LegH (HTTM-domain protein) from *Streptomyces* sp. CT34 and CrdG (HTTM-domain protein) from *C. lactis* RW3-42.

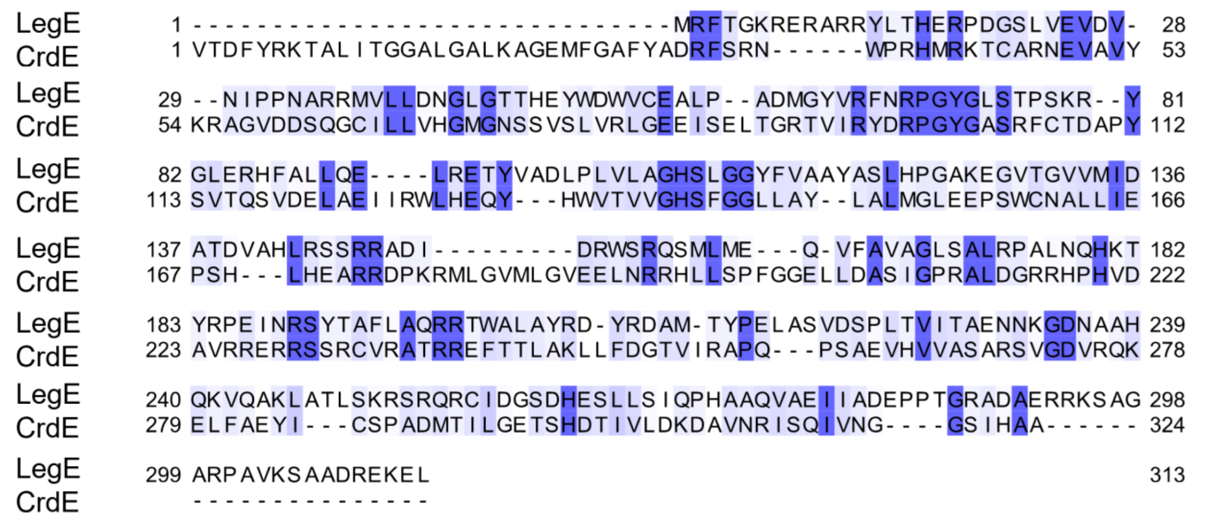

Figure S3: CLUSTALW sequence alignment of LegE (Alpha-beta hydrolase) from *Streptomyces* sp. CT34 and CrdE (Alpha-beta hydrolase) from *C. lactis* RW3-42.

```
LegF    1 VTVRVNTERLKTAAVTTSLAAWLVATAVAQMPEQ--RFDNLLKRGKLRIPTPNWRFFGP  57
CrdL    1 ---------MLRNVIEVVFGSWFTLTFLGQHPGGNHRSRGLLFRLSSTIFMPNWAFFAP  50

LegF   58 NPGVKDNHLLYRDVTDGKPGEWQEIPITRD---RAWYALAWNARNRSPKALFDAVQDVY 113
CrdL   51 NPGVYDDHLFYRVREGNEFSPWKEVVTSRNDDGPAWLSPFYSGASRRSKGIIDIFSTLE 109

LegF  114 VRSAAYGTAMEPVVQSSGYQLLSGYIRHHLPHAEGASHSQFLVMYSYLAAPEARQIEPI 172
CrdL  110 SLASPTLSRTDCNVIQAGRQAIANFMVVNADEI-SPHTSEYEVMLVR-AAGYAQDEDPV 166

LegF  173 FVSREFPLEDEGVVQPEPYPVAA                                     195
CrdL  167 FYYR-FAVRNGIADAPH------                                     182
```

Figure S4: CLUSTALW sequence alignment of LegF (LinL protein) from *Streptomyces* sp. CT34 and CrdL (LinL protein) from *C. lactis* RW3-42.

```
LegF    1 -----------MLLITGVLLGLLGVGASLTQPWAIGKLIEAAGKGESLAFPIVRLVGLF  48
CrdL    1 MTAFNLLRNNAGLITLGILLSLLSTAVTLFQPALVGQLISGVSSGELQQ-PLMLLLLTV  58

LegF   49 CLGAVFSALQAYVIGRAGENIVYDVRQVLTGRLLRADLTEFGKRPQGDILTRTVTDTSL 107
CrdL   59 LGTSVLTAATMYVVSIAADRTVRDMRKKITNHLLYLRVRELEKGGSGSFTTRVTSDTSI 117

LegF  108 VKIALSQSLAQLIVSGATVIGGVVLMFLIDVRLMLITMGCLGVASLLSLSIARKLRRVA 166
CrdL  118 VSTAFSSTLTDFVGGFTVIIGALIYMAVVDWKLLLVVIAVLLVALAIIVSISSSLQNLS 176

LegF  167 VQNRDDTGEFGTAVQRVLAALPTVKASRAETRETARIGRLAERARRSGIRVNAYNALFM 225
CrdL  177 SKVQDHLAALGEILQSALSAIRTIKAFRVETKVIGNLSAEIDHAYRNRRRMSFVEAVLE 235

LegF  226 PSMNVGTQGALAVVVGAGMAWVARGEMNMADLTTFIMYLFQLVSPLVTFFMGVGQFIQG 284
CrdL  236 PLSTVASYMALLAVVLFGSIRLSNGDLSGEALTVFVTALFLMLAPIVQVSQSLGAFFEA 294

LegF  285 RAAIQRVDELAQMPQEGNGAQAARSSVPIGPLPQHLPAVEFHDVRFGYRRGEEGERTQV 343
CrdL  295 RGALDRINQLLRLEVEDSTE--SSSSLCTGPT--PLGTIEFDAVSYVR------EKRTI 343

LegF  344 LHGISLTVPARGLTAVVGPSGAGKTSMFQLIERFYALDGGVILLGGQNIGTLPLDTVRG 402
CrdL  344 LDNATVTVKSGEKVALTGASGSGKTSILSLLLKFYDVSAGHIRIGGKDLEDWNRRDLRA 402

LegF  403 LVGYVQQDSATMRGTVRENLTYAHPHASEDDIWEA-VEMAGLTSVVANLPKGLDTELGD 460
CrdL  403 MVTYVEQEPDLLSGTLRENLILGTGESFDDETLIAMLGQFGLE--NFASVDGLSRSVGS 459

LegF  461 QGSGLSGGQRQRLCIARTLLQKPAVMLLDEATSNLDSDSERDFRDVLRRVSQQCAVIAI 519
CrdL  460 GNSGLSGGERQRVAIIRAVLQNAPVILVDEPTSALDSASAELSMSRL--LETDSTVIFT 516

LegF  520 AHRISTVVDAEKIVVLEGGRVRATGVHRELMEHDELYRRLAGSQLHAGAAPVATATHWV 578
CrdL  517 SHDANIVNLAERTLVVVDGRIVENSPNSRKSSNA------------------------- 550

LegF  579 SGEPGAGPLASALGWHQWGSGLNEQTVRLRPIRIEEQW                      616
CrdL      --------------------------------------
```

Figure S5: CLUSTALW sequence alignment of LegB (ABC transporter) from *Streptomyces* sp. CT34 and CrdT (ABC transporter) from *C. lactis* RW3-42.

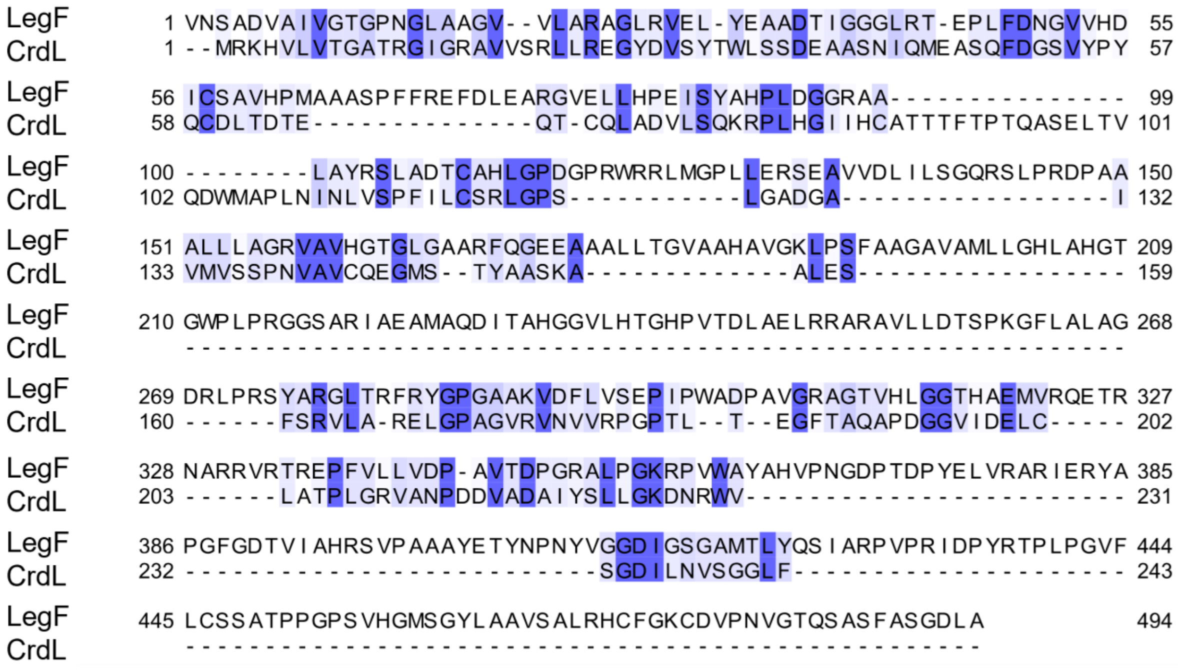

Figure S6: CLUSTALW sequence alignment of LegC (SDR oxidoreductase) from *Streptomyces* sp. CT34 and CrdC (SDR oxidoreductase) from *C. lactis* RW3-42.

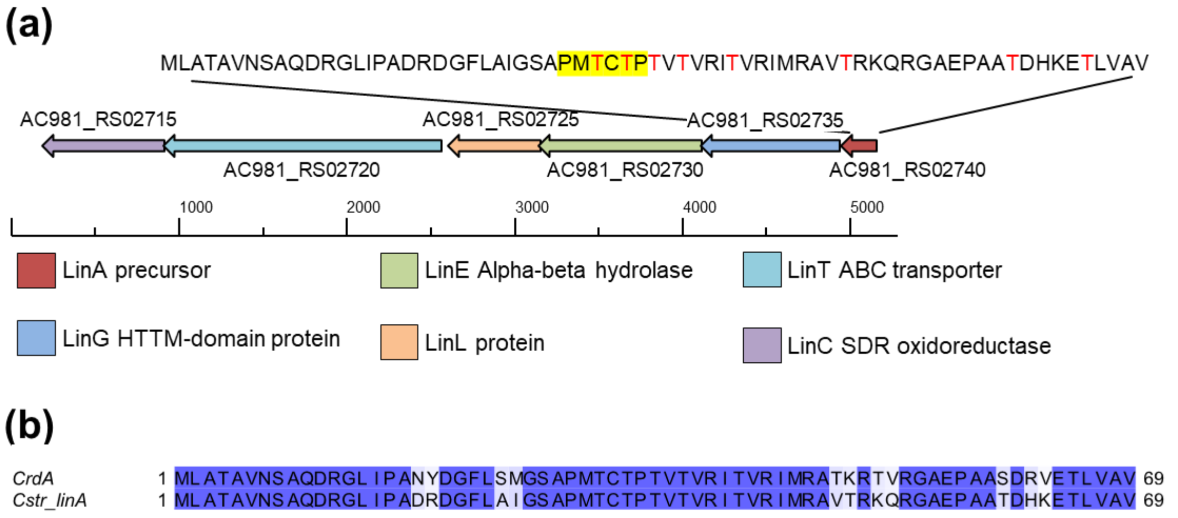

Figure S7: Putative linaridin gene cluster in *C. striatum* 1329_CAUR. (a) Gene cluster and annotation according to BLASTP analyses. (b) Sequence alignment of the *C. striatum* precursor peptide AC981_RS02740 (Cstr_linA) and corynaridin (CrdA) of *C. lactis*.

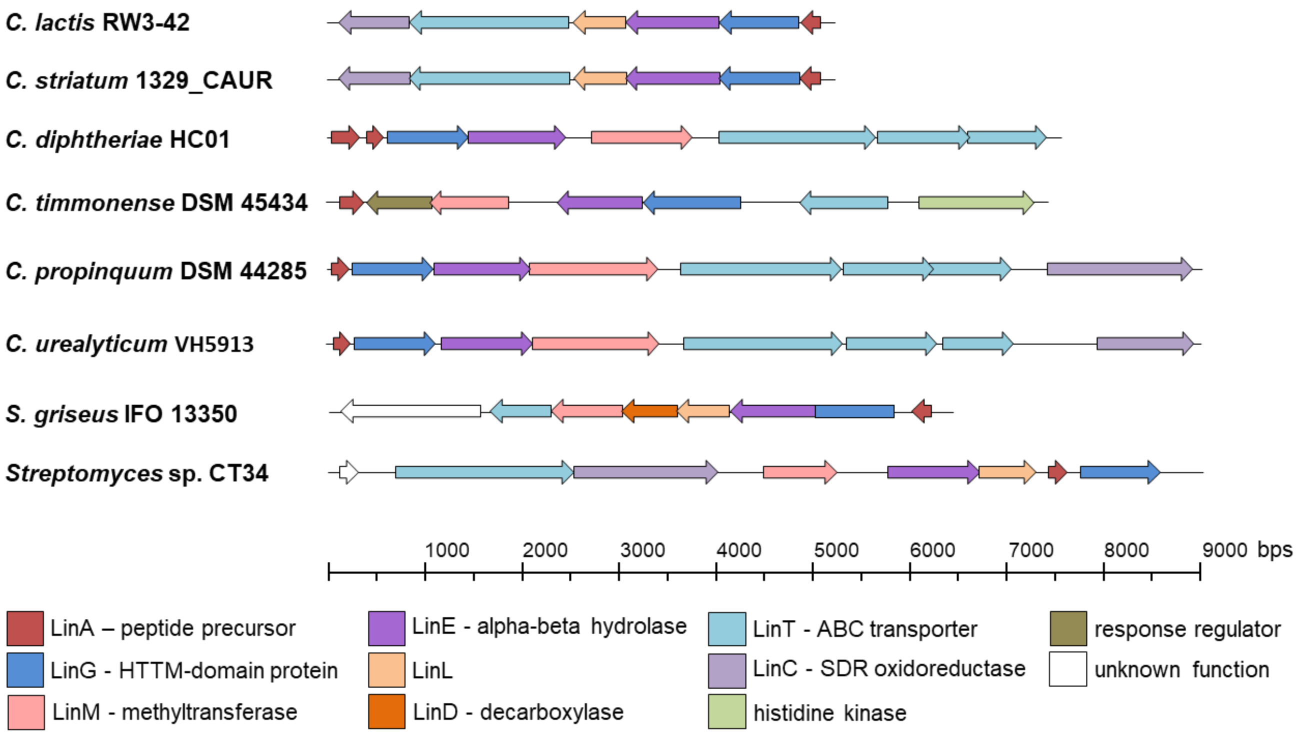

Figure S8: Putative linaridin gene clusters in *Corynebacterium* strains (1) compared with BGCs for other bacteriocins of the linaridin family. Gene cluster and annotation according to BLASTP analyses.

C. lactis **RW3-42**	MLATAVNSAQDRGLIPANYDGFLSMGSAPMTCTPTVTVRITVRIMRATKRTVRGAEPAASDRVETLVAV
C. striatum **1329_CAUR**	MLATAVNSAQDRGLIPADRDGFLAIGSAPMTCTPTVTVRITVRIMRAVTRKQRGAEPAATDHKETLVAV
C. diphtheriae **HC01**	MQPFSGFSLSSDERLAYFSIGHFILFQTTFQGVDLSALSQLVNDNTAFRGRLDGMAETALPPVTATPAIAVTAAKAAGAVIGGGAVSGAAYAAYRAVIK
	MSRLSTLVNNDQITLEQVHSPVMATPAAFGAGVVAGAKACGALVAAAGVGAAIASATKK
C. timmonense **DSM 45434**	MRNNITKFADEADLQQVGSLYGDSEPVLATIITTTITATTATTATTAAGYHSSRPDEVMAGIDEDAPVSEMLAARKDAMLV
C. propinquum **DSM 44285**	MSRLSTLVNNDQITLEQVHSPVMATPAAFGAGVVAGAKACGALVAAAGVGAAIATATKE
C. urealyticum **VH5913**	MSRLSTLVNNDQITLEQVHSPVMATPAAFGAGVVAGAKACGALVAAAGVGAAIASATKK
S. griseus **IFO 13350**	MRSEMTLTSTNSAEALAAQDFANTVLSAAAPGFHADCETPAMATPATPTVAQFVIQGSTICLVC
Streptomyces **sp. CT34**	MSVLAEFANTELVDVEPGRLGSEATPTMITPLATLATPEATPVGFAATSATAAAVNMITHDVTRH

Figure S9: Predicted precursor peptide sequences in *Corynebacterium* strains (1) compared with BGCs for other bacteriocins of the linaridin family. Threonine residues are displayed in red, serine residues in blue letters. The predicted hexapeptide cleavage site PxxxTP is highlighted in yellow.

Table S3: Results of cross-streak and spot-on-lawn assays with different indicator bacteria. Cross streak assay with *C. lactis* RW3-42 streaked in the middle of a BHI agar plate and indicator bacteria lateral to it. Nisin (250 µg/mL) and corynaridin RPC-fraction (>300 µg/mL) were spotted in 10 µl drops onto the plates. Spots were documented using an iBright imaging device or a light-table and camera. n.d. = not detected.

Strain	Cross streak	Nisin	Corynaridin
Bacillus subtilis DSM 402	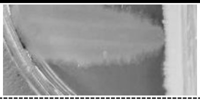	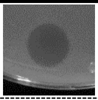	n.d.
Corynebacterium ammoniagenes DSM 20306	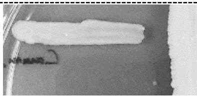	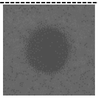	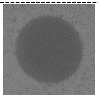
Corynebacterium amycolatum DSM 6922	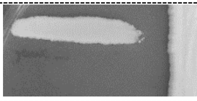	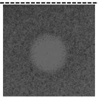	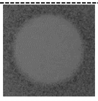
Corynebacterium canis DSM 45402	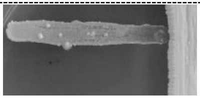	n.d.	n.d.
Corynebacterium casei DSM 44701	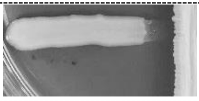	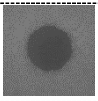	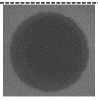
Corynebacterium efficiens DSM 44549	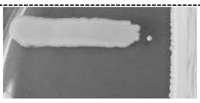	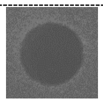	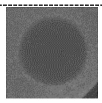
Corynebacterium glutamicum ATCC 13032	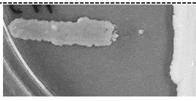	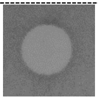	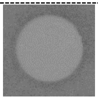
Corynebacterium lipophiloflavum DSM 44291	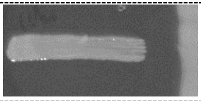	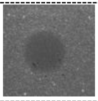	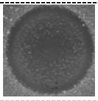
Corynebacterium striatum DSM 20668	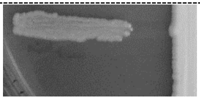	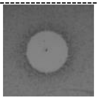	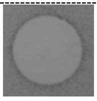
Corynebacterium xerosis DSM 20743	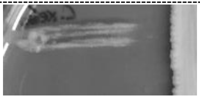	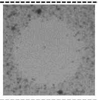	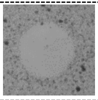
Cutibacterium acnes DSM 16379	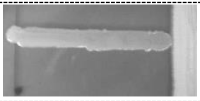	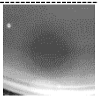	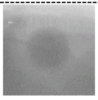
Escherichia coli K12 MG1655	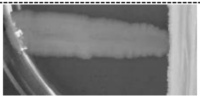	n.d.	n.d.

➔ continued

Table S3: continued.

strain	cross streak	nisin	corynaridin
Lactobacillus plantarum DSM 1055	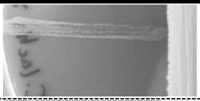	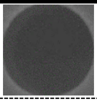	n.d.
Lactococcus lactis IL1403	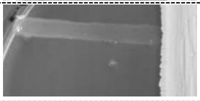	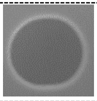	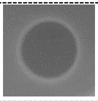
Listeria innocua LMG2785	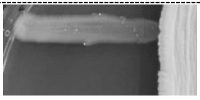	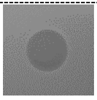	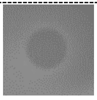
Listeria monocytogenes EGD-e	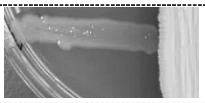	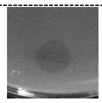	n.d.
Micrococcus luteus DSM 20030	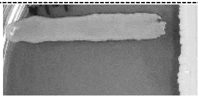	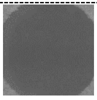	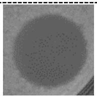
Pediococcus acidilactici 347	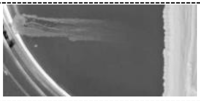	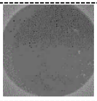	n.d.
Pseudomonas fluorescens DSM 50090	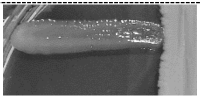	n.d.	n.d.
Staphylococcus aureus ATCC 29213	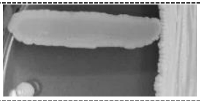	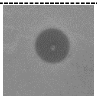	n.d.
Staphylococcus epidermidis DSM 3269	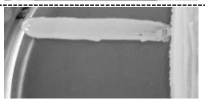	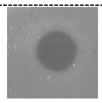	n.d.

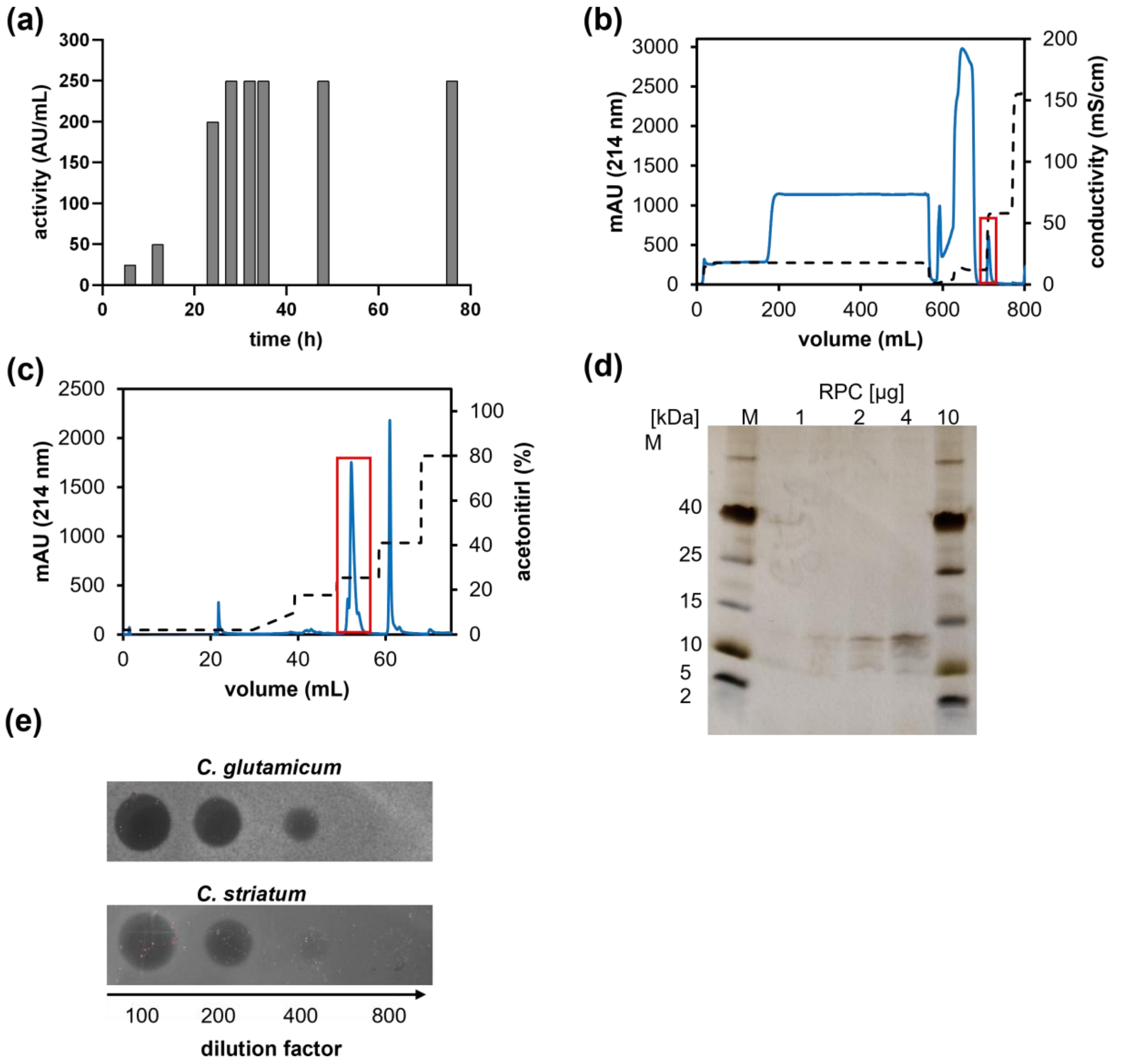

<u>Figure S10</u>: Improved procedure to obtain corynaridin in higher purity. (a) Activity over time of *C. lactis* supernatants after growth in CLIV minimal medium with 1 % (w/v) glucose, tested via spot on lawn assay. Values represent mean and standard deviation of three biological replicates. (b and c) Purification of corynaridin from supernatants obtained after growth of *C. lactis* RW3-42 in CLIV minimal medium by ion exchange chromatography (b) and subsequent reversed phase chromatography (c). Red boxes indicate fractions that were used for further experiments. (d) Silver stained Novex™ 10-20% TRIS Tricine gel loaded with different amounts of RPC-purified corynaridin. (e) Activity of the RPC purified fraction against *C. glutamicum* and *C. striatum*. The pooled fractions were vacuum dried and resuspended in H_2O (HPLC grade) to a concentration of 200 µg/mL.

<u>Table S4:</u> Complete list of all corynaridin variants with different number of dehydrated serine/threonine residues identified by LC-MS. Variants were considered identified if mass spectra for at least three charges with four isotopes had minimum signal intensities (counts per second; cps) of 10^3.

Peptide				
CT(Dhb)PT(Dhb)VT(Dhb)VRIT(Dhb)VRIMRAT(Dhb)KRT(Dhb)VRGAEPAAS(Dhas)DRVET(Dhb)LVAV				
**# dehydrated Ser/Thr**	**M_{mi} [Da]**	**Peaks**		
1	4087.26	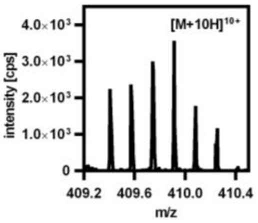	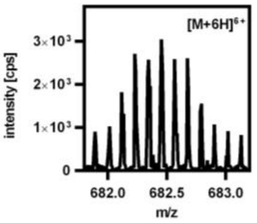	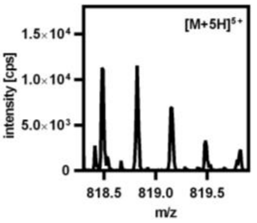
2	4069.25	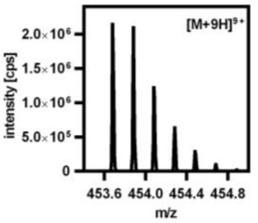	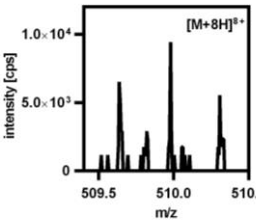	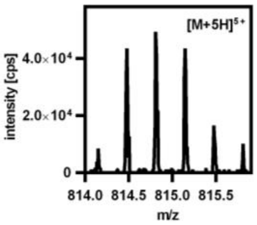
3	4051.24	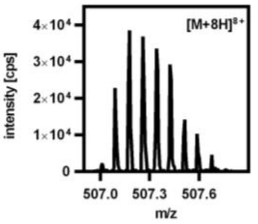	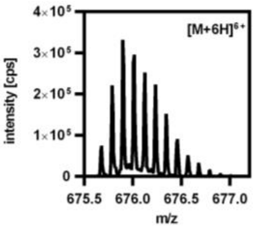	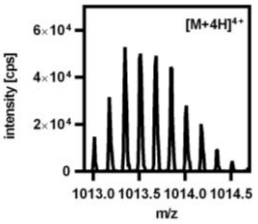
4	4033.23	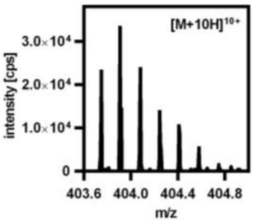	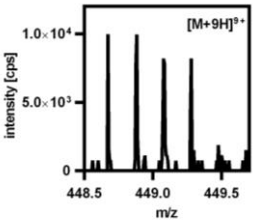	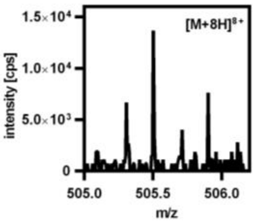
6	3997.21	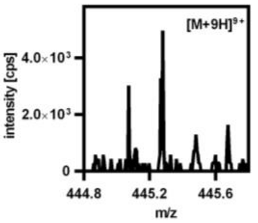	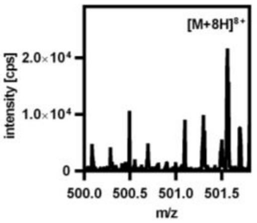	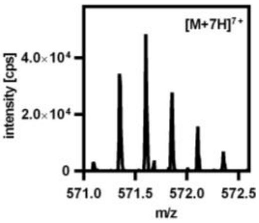
7	3979.20	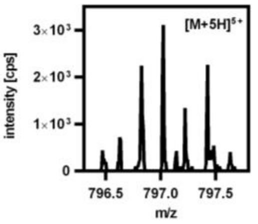	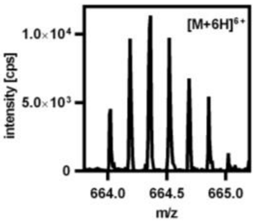	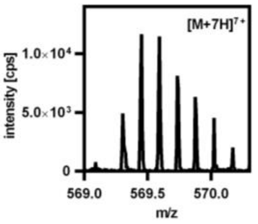
8	3961.19	See manuscript main text, Figure 5		

References

1. Georgiou MA, Dommaraju SR, Guo X, Mast DH, Mitchell DA. 2020. Bioinformatic and reactivity-based discovery of linaridins. ACS Chem Biol 15:2976–2985.
